# Identification and Comprehensive Evaluation of Resistant Weeds Using Unmanned Aerial Vehicle-Based Multispectral Imagery

**DOI:** 10.3389/fpls.2022.938604

**Published:** 2022-07-05

**Authors:** Fulin Xia, Longzhe Quan, Zhaoxia Lou, Deng Sun, Hailong Li, Xiaolan Lv

**Affiliations:** ^1^College of Engineering, Anhui Agricultural University, Anhui, China; ^2^College of Engineering, Northeast Agricultural University, Harbin, China; ^3^Institute of Agricultural Facilities and Equipment, Jiangsu Academy of Agricultural Sciences (JAAS), Jiangsu, China

**Keywords:** atrazine-resistant weed, multispectral reflectance, vegetation indices (VIs), unmanned aerial vehicle (UAV), deep convolutional neural networks (DCNNs)

## Abstract

Atrazine is one of the most widely used herbicides in weed management. However, the widespread use of atrazine has concurrently accelerated the evolution of weed resistance mechanisms. Resistant weeds were identified early to contribute to crop protection in precision agriculture before visible symptoms of atrazine application to weeds in actual field environments. New developments in unmanned aerial vehicle (UAV) platforms and sensor technologies promote cost-effective data collection by collecting multi-modal data at very high spatial and spectral resolution. In this study, we obtained multispectral and RGB images using UAVs, increased available information with the help of image fusion technology, and developed a weed spectral resistance index, WSRI = (RE-R)/(RE-B), based on the difference between susceptible and resistant weed biotypes. A deep convolutional neural network (DCNN) was applied to evaluate the potential for identifying resistant weeds in the field. Comparing the WSRI introduced in this study with previously published vegetation indices (VIs) shows that the WSRI is better at classifying susceptible and resistant weed biotypes. Fusing multispectral and RGB images improved the resistance identification accuracy, and the DCNN achieved high field accuracies of 81.1% for barnyardgrass and 92.4% for velvetleaf. Time series and weed density influenced the study of weed resistance, with 4 days after application (4DAA) identified as a watershed timeframe in the study of weed resistance, while different weed densities resulted in changes in classification accuracy. Multispectral and deep learning proved to be effective phenotypic techniques that can thoroughly analyze weed resistance dynamic response and provide valuable methods for high-throughput phenotyping and accurate field management of resistant weeds.

## Introduction

Weeds are one of the major factors affecting crop growth and are the most significant contributors to yield loss globally (Quan et al., 2021). Overreliance on commonly used chemical herbicides has resulted in the appearance of several herbicide-resistant weed biotypes (Colbach et al., 2017). Developing a method that can indicate herbicide resistance within an acceptable timeframe after an application can potentially help growers manage their fields more effectively (Krähmer et al., 2020).

Atrazine (chemical name: 2-chloro-4-ethylamino-6-isopropylamino-1,3,5-triazine) belongs to the S-triazine class of herbicides and blocks the electron flow between photosystems (Foyer and Mullineaux, 1994). Atrazine herbicide can significantly reduce photosynthesis by reducing photosystem II (Sher et al., 2021) and is a widely used herbicide in maize fields to control broadleaf and grassy weeds (Williams et al., 2011). Its widespread use has also accelerated the evolution of weed resistance mechanisms (Kelly et al., 1999; Williams et al., 2011; Perotti et al., 2020).

However, high-throughput herbicide resistance phenotyping remains a technical bottleneck, limiting the ability to effectively manage weeds in the field. Before herbicide application, there is no significant difference in the visual appearance of susceptible and resistant weeds of the same species (Eide et al., 2021a). Laboratory determination of various enzymes present within plant leaves can identify atrazine resistance but is impractical to use in large-scale applications (Liu et al., 2018). Hyperspectral systems to detect differences between resistant and susceptible biotypes have shown potential in controlled environments (Shirzadifar et al., 2020b), but their effectiveness is drastically reduced once introduced into field conditions (Shirzadifar et al., 2020a). The unstable performance of thermal imagery further suggested that canopy temperature data were likewise not a reliable predictor of weed resistance (Eide et al., 2021b). Outdoor resistance identification methods include whole-plant dose–response assay tests (Huan et al., 2011), but their investigation area is fixed and limited, resulting in high deployment expense and poor timeliness. Thus, current phenotypic analysis methods can hardly satisfy the high-throughput survey requirements for resistant weeds in the field.

Field-based fast, accurate, and robust phenotyping methods are essential for atrazine-resistant weed investigation. Atrazine applications reduce the efficiency of the photosynthetic mechanism and affect chlorophyll and other pigments, which change the spectral reflectance of plants in the visible/near-infrared range (Sher et al., 2021). Therefore, it is assumed that the spectral characteristics of susceptible weeds should show different pathways compared to resistant weeds after herbicide application. These physiological changes induced by herbicide stress have laid the foundation for monitoring resistance using vegetation indices (VIs) (Duddu et al., 2019). Multispectral bands and the normalized difference vegetation index (NDVI) provide improved glyphosate resistance classification (Eide et al., 2021a). Therefore, Vis-based high-throughput phenotyping methods can be reliably applied to atrazine-resistant weed investigation in the field.

Unmanned aerial vehicles (UAVs) are a popular remote sensing platform successfully used to obtain high-resolution aerial images for weed detection and mapping (Su et al., 2022) because they can be equipped with various imaging sensors to collect high-spatial, -spectral, and -temporal resolution images (Yang et al., 2017, 2020). For example, UAVs have been used for physiological and geometric plant characterization (Zhang et al., 2020; Meiyan et al., 2022), as well as for pest and disease classification (Dai et al., 2020; Xia et al., 2021) and resistant weed identification (Eide et al., 2021a). In addition, remote sensing imagery is linked to specific farm problems through deep learning for the identification of biological and non-biological stresses in crops (Francesconi et al., 2021; Ishengoma et al., 2021; Jiang et al., 2021; Zhou et al., 2021), segmentation, and classification (He et al., 2021; Osco et al., 2021; Vong et al., 2021). These studies show that the combination of UAV remote sensing and deep learning provides the scope for large-scale resistant weed evaluation (Krähmer et al., 2020; Wang et al., 2022).

This study explores the potential for using multispectral images collected by UAVs in crop fields for identifying resistant weeds and proposes an effective method to identify resistant weeds in real field environments. We propose a weed spectral resistance index called WSRI = (RE-R)/(RE-B) to investigate resistant weeds by analyzing the canopy spectral response of barnyardgrass and velvetleaf. The fusion of multispectral and RGB images combining canopy spectral and texture feature information and applying a deep convolutional neural network (DCNN) are carried out to evaluate the potential for identifying resistant weeds in the field based on their dynamic response.

## Materials and Methods

### Test Site and Experimental Setup

**FIGURE 1:**
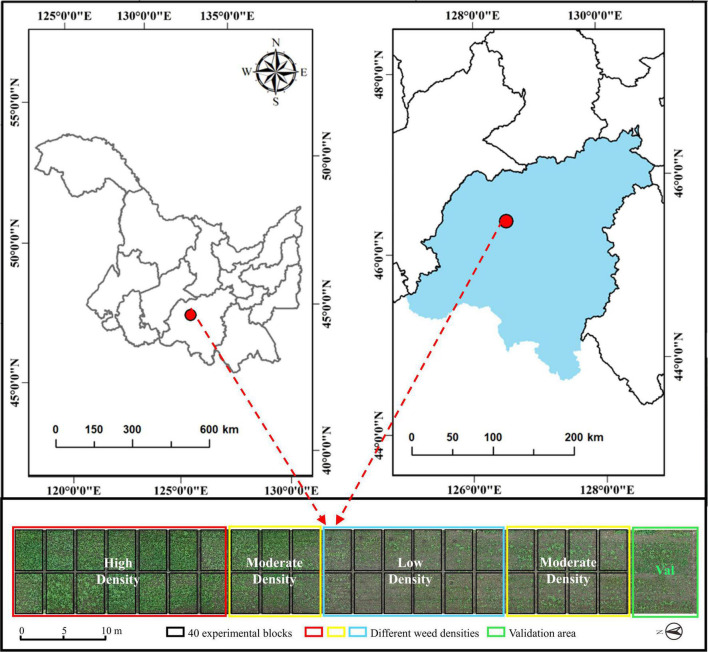
Distribution of test sites and test fields.

The weed resistance experiment was conducted at the Xiangyang Farm, Northeast Agricultural University, Harbin, Heilongjiang, China (45°61′ N, 126°97′ E), as shown Figure 1. The region has a cold-temperate continental climate, with average annual precipitation of 400–600 mm and an average annual effective temperature of 2,800°C. The experimental soil type is black soil, with a soil tillage layer, a nitrogen content of 0.07–0.11%, a fast-acting phosphorous content of 20.5–55.8 mg/kg, and a fast-acting potassium content of 116.6–128.1 mg/kg.

Two different weed species were selected for this study. Common broadleaf and grassy weeds in the Heilongjiang region include barnyardgrass (Echinochloa crusgalli (L.) Beauv) and velvetleaf (Abutilon theophrasti Medicus). Weed seeds were collected from 20 different fields in Heilongjiang and confirmed to be atrazine-susceptible and -resistant biotypes (Liu et al., 2018). The seeds were air-dried and stored at 4°C. The field was treated with glufosinate at 0.45 kg active ingredient (AI) ha^–1^ plus pendimethalin at 1.12 kg AI ha^–1^ before planting to kill existing vegetation and provide residual weed control 1 week before crop planting.

In this trial, maize seeds were first sown in black soil on May 13. The weed seeds were mixed with sand, dropped on the soil surface, and then harrowed immediately after maize sowing. Weed seed dropping is divided into three densities (low, 40 seeds m^–2^; moderate, 160 seeds m^–2^; high, 320 seeds m^–2^). After maize germination, slight spray irrigation was applied to the whole field to accelerate weed germination. The herbicide atrazine (Ji Feng Pesticide Co., Jilin, China) was then sprayed at a uniform rate on 1st June when the maize reached the three-leaf stage.

**FIGURE 2:**
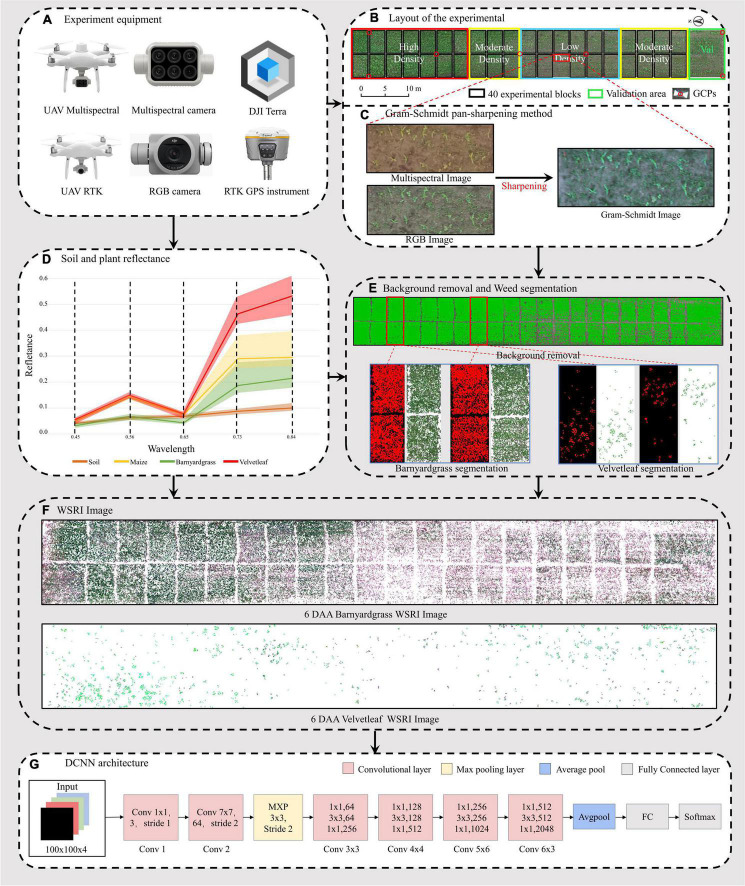
Workflow of the unmanned aerial vehicle (UAV) high-throughput field weed resistance approach. **(A)** DJI Phantom 4 Multispectral, DJI Phantom 4 RTK, DJI Terra, and RTK GPS instrument for collecting field images. **(B)** Digital orthophoto maps (DOM) of three maize field densities (low, moderate, high) for weed resistance research. **(C)** Gram–Schmidt sharpening for improving spectral image information. **(D)** Reflectance values of four objects in the orthophoto (soil, maize, barnyardgrass, and velvetleaf). **(E)** Soil and maize removal and two types of weed segmentation, including barnyardgrass and velvetleaf. **(F)** Two weed image datasets from 6 days after atrazine application (6 DAA) used in the classification models. **(G)** Deep convolutional neural network (DCNN) architecture.

In the experimental field, 40 plots were divided into three weed density treatments (Figure 2B). Each treatment consisted of 12 or 14 plots measuring 3 m × 5 m in six rows with a 0.6-m row spacing. A 1-meter-wide protection plot surrounded the entire field to reduce edge effects. This study investigated the ground truthing data before the atrazine application day. The manual measurements for ground truthing consisted of the survival status of the two weed types and geographical coordinates after application.

### Data Acquisition

#### Unmanned Aerial Vehicle Image Collection

Multispectral and RGB images were collected with DJI Phantom 4 Multispectral and DJI Phantom 4 RTK UAVs (SZ DJI Technology Co., Ltd., Shenzhen, China), as shown in Figure 2A. The UAVs are equipped with centimeter-level navigation and positioning systems. The DJI Phantom 4 Multispectral camera simultaneously acquires images in blue (B), green (G), red (R), red edge (RE), and near-infrared (NIR) bands (Table 1) at a 1600 × 1300 pixel resolutions. The DJI Phantom 4 RTK has a camera with an FC6310R lens (*f* = 8.8 mm) and a 4864 × 3648 pixel resolution. Based on a UAV flight test with manually controlled height varying from 10 to 30 m above ground, the UAV altitude was finally set to 15 m with no disturbance to the leaves. The ground sampling distances (GSDs) of multispectral and RGB images were 0.79 and 0.41 cm pixel^–1^, respectively. UAV flights were conducted in the field on 6th and 20th May 2021 to collect the early season information needed for the study. RGB images were acquired first, and then multispectral images were acquired each day. The mean forward overlap of the photographs was 80%, and the mean sidelap was 70%. The UAV observations covered the complete experimental range (Table 2). However, some data are missing because of weather conditions.

**TABLE 1:** Multispectral camera band specifications.

Band	Name	Center wavelength (nm)	Bandwidth (nm)
1	Blue	450	32
2	Green	560	32
3	Red	650	32
4	Near infrared	840	52
5	Red edge	730	32
			

**TABLE 2:** Weather conditions during data collection.

Band**	Collection Date	Air Temp (°C)	Weather
BAD	2021.05.30	11∼22°C	Clear day
AD	2021.06.01	11∼22°C	Cloudy day
1 DAA	2021.06.02	13∼21°C	Cloudy day
2 DAA	2021.06.03	10∼20°C	Cloudy day
4 DAA	2021.06.05	10∼18°C	Cloudy day
5 DAA	2021.06.06	11∼21°C	Clear day
6 DAA	2021.06.07	12∼25°C	Clear day
7 DAA	2021.06.08	13∼28°C	Clear day
8 DAA	2021.06.09	18∼27°C	Cloudy day
10 DAA	2021.06.11	15∼28°C	Clear day
14 DAA	2021.06.15	18∼29°C	Cloudy day

^**^BAD, before atrazine application day. AD, atrazine application day; DAA, days after atrazine application.

#### Image Preprocessing

Approximately 2,000 images per flight were used for the photogrammetry process using DJI Terra software (SZ DJI Technology Co., Ltd., Shenzhen, China) to obtain images of the entire experimental area. The global navigation satellite system (GNSS) real-time motion control measured seven ground control points (G) to obtain accurate geographical references. The seven GCPs were measured with a GNSS real-time kinematic (RTK) receiver (RTK GPS instrument i50, CHC Navigation Co., Ltd., Shanghai, China). The reflectance correction and radiometric calibration use a 3 m^2^ carpet reference and the Spectron on software (Resonon Inc., Bozeman, MT, United States). The empirical line method was then used to convert the image’s digital number (DN) value to a reflectance value (Figure 2D).

### Development of Specific Indices Identifying Atrazine-Resistant Weeds

Canopy spectral reflectance differs between weed species, and some spectrum regions may better identify atrazine resistance status. Sample selection was based on the weed survival 14 days after application. The reflectance of susceptible and resistant biotypes of two weed species was counted in the multispectral images after 2 days of application.

**FIGURE 3:**
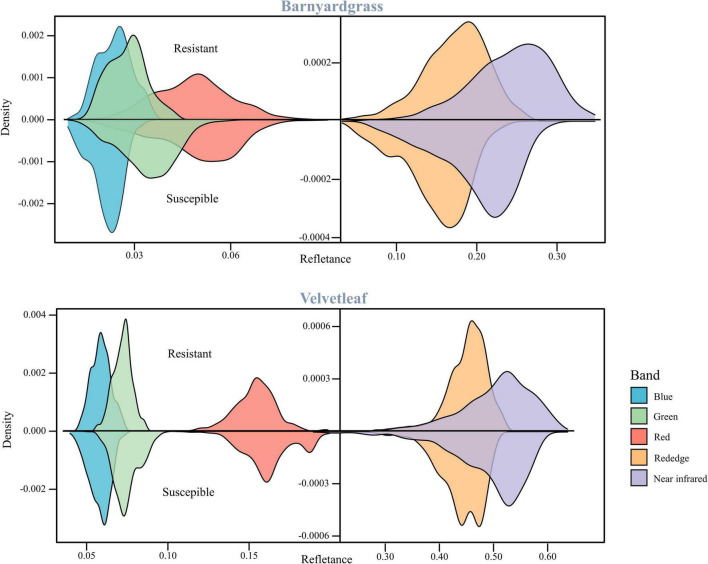
Reflectance density maps of two weed resistant and susceptible biotypes.

Figure 3 shows barnyardgrass and velvetleaf reflectance density maps for five bands extracted from multispectral images of susceptible and resistant biotype regions. Slight differences between susceptible and resistant biotypes were observed in the green, red, red edge, and near-infrared bands, and the differences between the red (650 nm) and red edge (780 nm) bands show greater stability (Jin et al., 2020). Part of the blue (450 nm) band was observed to reduce the differences in leaf surface reflectance, thereby improving the correlation between the vegetation index and leaf pigment content (Sims and Gamon, 2002). Therefore, we proposed a weed spectral resistance index named WSRI = (RE-R)/(RE-B) to calculate and evaluate actual field environmental resistant weeds and tested it in this study (Figure 2F).

Many VIs have similar effects when dealing with classification problems, differing in their index form expressions. Simple vegetation index forms, such as the NDVI and ratio vegetation index (RVI), are universal to the problem and reflect vegetation information well in many cases. In this study, we entered our multispectral image data into nine previously published VIs (Table 3) and the WSRI to evaluate and compare their weed resistance classification accuracies.

**TABLE 3:** Vegetation indices used in this study.

Category	Features	Expression**	References
DVI	Difference vegetation index	NIR-R	Jordan, 1969
MTCI	MERIS terrestrial chlorophyll index	(NIR-RE)/(RE-R)	Dash and Curran, 2004
NDVI	Normalized differential vegetation index	(NIR-R)/(NIR + R)	Tucker et al., 1979
GNDVI	Green normalized difference vegetation index	(NIR-G)/(NIR + G)	Gitelson and Merzlyak, 1998
NDRE	Normalized difference red-edge index	(NIR-RE)/(NIR + RE)	Sims and Gamon, 2002
RENDVI	Red-edge normalized difference vegetation index	(RE-R)/(RE + R)	Sims and Gamon, 2002
RVI	Ratio vegetation index	NIR/R	Birth and Mcvey, 1968
RERVI	Red-edge ratio vegetation index	NIR/RE	Vincini and Frazzi, 2009
PSRI	Plant senescence reflectance index	(R-G)/NIR	Merzlyak et al., 1999
WSRI	Weed Spectral Resistance Index	(RE-R)/(RE-B)	This paper

^**^B, G, R, RE, and NIR represent blue, green, red, red-edge, and near-infrared bands, respectively.

### Image Fusion

The multispectral images with low spatial resolution used for classification lost almost all texture features. However, susceptible and resistant biotype differences are expressed in the texture information. The high spatial resolution of RGB images compensated for the lost texture information in the multispectral images, so image fusion using the Gram–Schmidt pan-sharpening method in ENVI 5.4.1 (EXELIS, Boulder, CO, United States) was used (Figure 2C). The fusion images have five bands: blue, green, red, red-edge, and near-infrared.

The Gram–Schmidt pan-sharpening method is based on Gram–Schmidt (GS) orthogonalization. GS orthogonalization is performed to orthogonalize matrix data or digital image bands (Laben and Brower, 2000). It first created a simulated low-resolution panchromatic band as a weighted linear combination of multispectral bands. Then, GS orthogonalization is performed using all bands, including the simulated panchromatic and multispectral bands. The simulated panchromatic band is the first band in GS orthogonalization. After making all bands orthogonal by using GS orthogonalization, the high-spatial resolution panchromatic band replaces the first GS band. Last, an inverse GS transform creates the pan-bands (Laben and Brower, 2000; Ehlers et al., 2010).

### Background Removal and Weed Segmentation

Because of the reflectance differences between soil and plants (Figure 2D), Otsu’s thresholding algorithm (Ostu et al., 1979) was used to separate vegetation from the soil, find an optimal value to be used for segmentation, and then adjust the threshold value, if necessary, to improve separation of the plants from the soil (Figure 2E; Liao et al., 2020).

Manual segmentation of maize and weeds has higher accuracy but is expensive and time-consuming. UAV multispectral and RGB images were segmented for maize, barnyardgrass, and velvetleaf using the support vector machine (SVM) classifier (Cortes et al., 1995). The four-leaf stage of maize did not shade the weeds significantly and separated the maize and weeds better. A binary mask layer was created to segment the maize and the two weed types from the UAV images’ extracted spectral and texture features for further processing (Figure 2E). The binary mask layer was generated in ENVI based on manually tagged template data.

The performance of the SVM classifier was evaluated using the confusion matrix and accuracy statistics, with the overall accuracy based on randomly selected independent test samples. The overall accuracy of SVM classification is 94.4%, which meets the experimental requirements. The zonal statistics were obtained using ArcPy and the Python 2.7 programming language to remove soil and maize and segment the barnyardgrass and velvetleaf.

### Dataset Production

**FIGURE 4:**
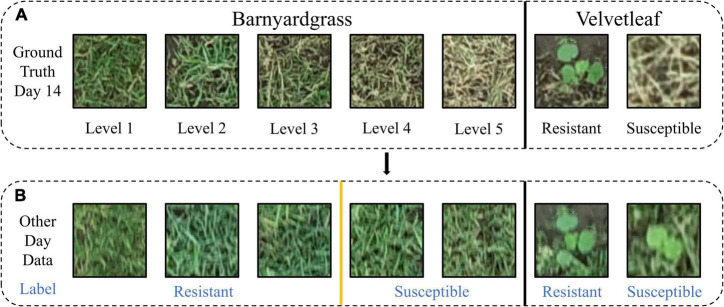
Two weeds belonging to blocks were evaluated as susceptible and resistant. **(A)** Manual resistance level and label based on weed death coverage 14 days after atrazine application. **(B)** Visualization of the data labels on other days.

Different application effects were observed in the experimental area, and a training template was created for individual velvetleaf plants based on survival status 14 days after application (Figure 4A). The training template contained two classes: susceptible velvetleaf and resistant velvetleaf (Figure 4B).

Because barnyardgrass grows densely and is mostly aggregated, it is not easy to separate them into individual plants (Maun and Barrett, 1986). In this study, the resistance level was set according to the death rate of barnyardgrass in the same area 14 days after application. Figure 4A shows the example plants from blocks at different resistance levels (example of barnyardgrasses in high-density areas). Resistance level 1 is defined as 0–25% death of barnyardgrasses; resistance level 2 is 26–50% death of barnyardgrasses; resistance level 3 is 51–75% death of barnyardgrasses; resistance level 4 is 76–95% death of barnyardgrasses; and resistance level 5 indicates an entirely dead barnyardgrass block. Blocks with resistance levels less than or equal to 3 were considered resistant (Figure 4B) because these blocks exceeded the threshold for weed control in farmland weeds (Anru and Cuijuan, 2014).

The image patch of each weed plot must be cropped from the barnyardgrass WSRI fusion segmentation image to build the dataset for DCNN modeling. Thus, a region of interest (ROI) shapefile was created in ArcMap 10.3 (Esri Inc., Redlands, CA, United States), and rectangles measuring around 0.5 m × 0.5 m were drawn. The cropped patch sizes were approximately 100 × 100 pixels. All data sets are four bands with a combination of WSRI images and RGB images.

The other UAV images throughout and after application were also processed to generate time-series image patches for dynamic weed resistance classification. Rotated image enhancement was applied to display the different shapes and directions of the weeds in the field. Four clockwise rotations (0°, the original data; 90°; 180°; and 270°) were performed for image enhancement. For the barnyardgrass dataset, the original 1,750 observations were increased four times, with 3,128 observations representing resistant blocks and 3,872 observations representing susceptible blocks for 7,000 observations each day and 28,000 total observations. For the velvetleaf dataset, the original 480 observations were increased four times, with 1,136 observations representing resistant plants and 784 observations representing susceptible plants for 1,920 observations each day and 7,680 total observations. Before the data augmentation, all data were randomly split into training and validation sets in an 8:2 ratio. The model performance was tested using a validation area (Figure 4B) to illustrate model’s the generality and robustness.

### Deep Convolutional Neural Network for Resistant Weed Classification

A DCNN (Figure 2G) for classifying resistant weeds was constructed using MATLAB R2021a (MathWorks Inc., Natick, MA, United States). The model was trained and tested on an NVIDIA 2080Ti GPU with 48-GB RAM and on a 64-bit Windows 10 operating system. CUDA version is 11.4.

The network was built based on the ResNet-50 model (He et al., 2016) and transfer learning (Kieffer et al., 2017). This study used the Resnet-50 model pre-trained on ImageNet (Krizhevsky et al., 2012) without fully connected (FC) layers for transfer learning. The input size was changed to 100 × 100 × 4 to match the size of the image patches. Then a convolutional layer (size of 3 × 3 × 3) was added behind the input 4-band images for reduced dimension on data. The ReLU activation layer was appended behind the convolutional layer to add non-linear characteristics. The dropout regularization method was deployed after the FC layer to reduce overfitting (Srivastava et al., 2014), and the dropout rate was set at 30%.

An Adam optimizer (Kingma and Ba, 2014) was used with a 10^–4^ learning rate and 10^–3^ decay to adaptively optimize the training process. The batch size was set to 128, and the data generator generated each batch with real-time data augmentation. The model was trained for 300 epochs with 10 batches per epoch. The accuracy of each classification was observed using a confusion matrix. Accuracy metrics were averaged from five repeats of randomized holdback cross-validation.

## Results

The DCNN was applied to classify weed resistance using the canopy spectral and textural information extracted from the UAV multispectral and RGB sensors, and the results are shown in Table 4.

**TABLE 4:** Resistant weed classification performance summary.

Species	Feature type	Metrics	2DAA	4DAA	6DAA	8DAA
Barnyardgrass	RGB	Accuracy	0.554	0.609	0.692	0.772
	WSRI		0.571	0.634	0.724	0.796
	DVI		0.533	0.583	0.641	0.717
	MCTI		0.526	0.559	0.619	0.693
	NDVI		0.556	0.591	0.654	0.746
	GNDVI		0.548	0.570	0.635	0.712
	NDRE		0.551	0.587	0.655	0.737
	NDVI-RE		0.564	0.597	0.657	0.759
	RVI		0.559	0.584	0.681	0.755
	RVI-RE		0.543	0.576	0.646	0.722
	PSRI		0.527	0.566	0.624	0.709
	5 BANDS		0.551	0.582	0.652	0.776
	WSRI + RGB		0.602	0.665	0.761	0.811
Velvetleaf	RGB	Accuracy	0.529	0.596	0.753	0.905
	WSRI		0.541	0.604	0.767	0.914
	DVI		0.532	0.573	0.691	0.867
	MCTI		0.526	0.562	0.677	0.822
	NDVI		0.547	0.578	0.705	0.894
	GNDVI		0.539	0.567	0.686	0.875
	NDRE		0.545	0.562	0.679	0.871
	NDVI-RE		0.528	0.583	0.711	0.907
	RVI		0.539	0.571	0.700	0.891
	RVI-RE		0.537	0.576	0.694	0.898
	PSRI		0.525	0.559	0.652	0.834
	5 BANDS		0.558	0.598	0.702	0.902
	WSRI + RGB		0.551	0.634	0.798	0.924

*DAA, days after application; WSRI, weed spectral resistance index.*

### Contribution of Spectral Bands and Vegetation Indices in the Resistant Weed Classification

The susceptible and resistant biotype reflectance densities of barnyardgrass and velvetleaf after atrazine application are shown in Figure 3. Spectral band differences between susceptible and resistant biotypes are related to the chlorophyll content and cell wall structure of the weed species.

Atrazine-resistant weed biotypes showed a slightly lower reflectance than susceptible weed biotypes in the visible light region. The differences between susceptible and resistant biotypes were more significant in the red edge and near-infrared regions, and the resistant biotypes showed increased spectral reflectance. These effects are related to the low chlorophyll content of susceptible biotypes, corresponding to plant stress response (Gomes et al., 2016). The main reason is that the application of atrazine reduces photosynthesis and destroys the pigments (Hess, 2000; Zhu et al., 2009). The red band is the central band of chlorophyll, which is the specific chlorophyll absorption band (Tros et al., 2021). The red edge position, which is the slope inflection point between red absorption and near-infrared reflectance, is usually used to correlate the chlorophyll content (Horler et al., 1983; Zarco-Tejada et al., 2019). Thus, the red and red edge bands are stable for the classifying atrazine-resistant weed biotypes.

**FIGURE 5:**
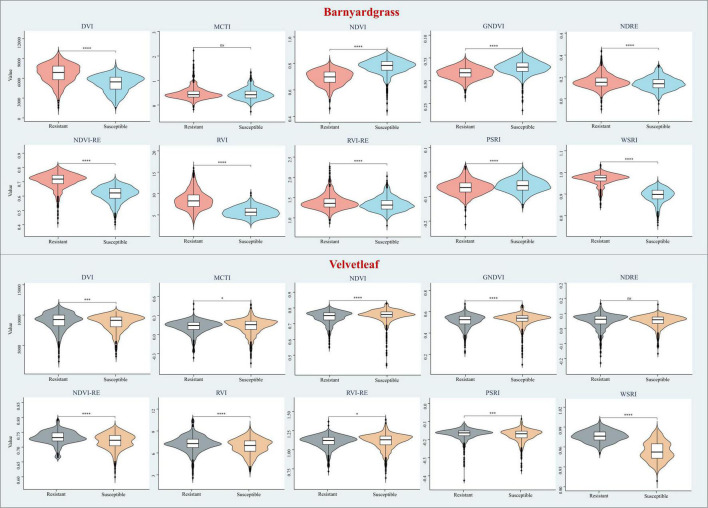
Violin plots of different vegetation indices for susceptible and resistant biotypes of barnyardgrass and velvetleaf 2 days after application, where *p < 0.05; **p < 0.01; ***p < 0.001; and ****p < 0.0001 indicate significant differences between the susceptible and resistant biotypes, and *ns* indicates no significance differences.

This study selected the most commonly used VIs to include some stress indices and compare their results from the DCNN with the WSRI (Table 4). Among these, stress and pigmentation changes resulted from atrazine herbicide application. To further explore the differences in the vegetation index distributions for observing susceptible and resistant biotypes, violin plots of barnyardgrass and velvetleaf for 10 VIs 2 days after application are shown in Figure 5.

The results in Figure 5 show that WSRI, DVI, NDVI, NDVI-RE, and RVI VIs distinguish between susceptible and resistant barnyardgrasses, with a common trait of these indices being that they all contain red bands. The difference vegetation index (DVI) and the RVI have little differences in susceptible and resistant biotypes because they do not integrate multi-band information well. The NDVI-RE used the red-edge bands to replace the NIR bands, resulting in a slightly better classification than the NDVI.

The WSRI retained the numerator structure of the NDVI-RE index and added the blue bands to the denominator to eliminate the spectral interference between pigments to achieve a better classification result. However, the WSRI classification of susceptible and resistant velvetleaf was very poor compared with barnyardgrass at the early stage of application, and the NDVI-RE and WSRI provided only partial classification. The WSRI makes the resistant weed data more concentrated and the susceptible weed data more dispersed, widening their differences. The average spectral response of barnyardgrass shows a more prominent separation than velvetleaf, possibly *via* lower herbicide uptake at the cuticular level, causing it to respond more slowly to herbicide stress (Couderchet and Retzlaff, 1995). In addition, velvetleaf has a higher reflectance than barnyardgrass, resulting in spectral changes that are more difficult to represent effectively.

### Contribution of Spectra and RGB in Resistant Weed Classification

Spectral information with the DCNN resulted in the highest weed resistance classification accuracy when using a single sensor. The single-band WSRI vegetation information surpasses the RGB texture information, even though the RGB image resolution is about 10 times higher than that of the spectral images. The difference in accuracy between them was largest 6 days after application, while the difference was smallest 8 days after application.

As shown in Table 4, the combination of WSRI spectral and RGB structural information improved accuracy, compared to using only a single sensor. RGB-derived detailed texture features, such as slight leaf discoloration and rolling, are not obtained from spectral features (Rischbeck et al., 2016; Stanton et al., 2017). In addition, canopy structure information can overcome the asymptotic saturation problems inherent to spectral features to some extent (Wallace, 2013; Maimaitijiang et al., 2020). Therefore, the combination of spectral and textural information improves classification accuracy. It should be noted that the accuracy improvement was not substantial, and combining multispectral and RGB information is likely attributed to information homogeneity and redundancy among canopy spectral and textural features (Pelizari et al., 2018; Maimaitijiang et al., 2020).

### Impacts of Different Times in Resistant Weed Classification

**FIGURE 6:**
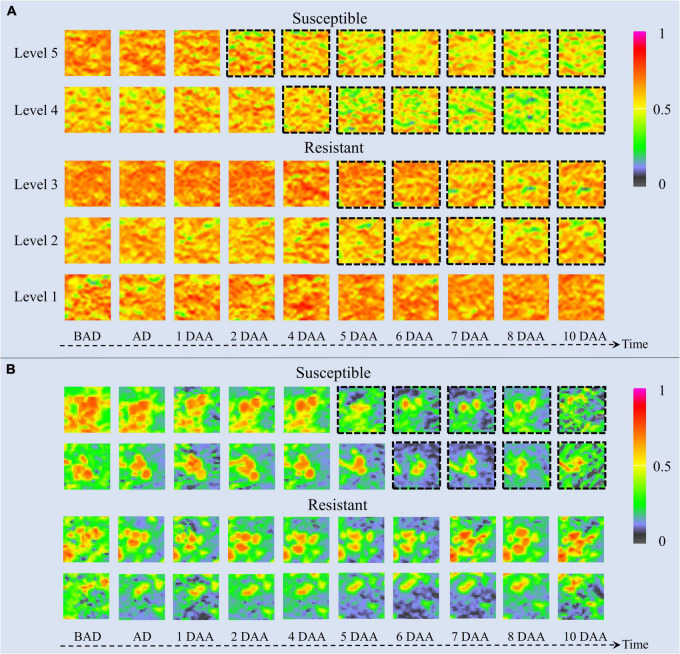
UAV-based visualization of two weed species with dynamic changes in NDVI images. **(A)** Image patches of five resistance levels of barnyardgrass under herbicide stress, where dashed black borders around the patches indicate significant changes. **(B)** Image patches of two susceptible and two resistant velvetleaf under herbicide stress, where dashed black borders around the patches indicate significant changes. BAD is before application day; AD is application day; DAA is days after application.

The time-series NDVI image patches of barnyardgrass and velvetleaf during the atrazine application stage are visualized in Figure 6. NDVI images reflect vegetation health status and nutrient information (Eide et al., 2021a). The color of the plant areas in the NDVI images represent the plant health status, where NDVI values close to 1 and redder plant regions mean healthier plants. As time increased, herbicide stress became more severe, and differences in resistance levels among weed blocks were increasingly evident. As shown in Figure 6A for barnyardgrass, the susceptible biotypes changed rapidly under herbicide application. About 3 days after application, the NDVI image of the leaves changed from red to yellow or even green, meaning that the vital characteristics of the susceptible biotypes gradually diminished.

By contrast, the resistant biotypes changed slowly with low amplitudes under the herbicide application. About 5 days after application, the NDVI image of the leaves changed slightly from red to yellow. The higher the resistance level, the smaller the change toward yellow. The highest resistance level showed only signs of stopping the growth and then completely recovered to normal growth about 4 days after application.

The change rate under herbicide stress conditions and the recovery speed after the application reflect resistance at different stages. The higher the resistance level of the barnyardgrass plots, the later the changes appeared. The large number of resistant barnyardgrasses surrounding susceptible barnyardgrasses made it difficult to observe changes in high-resistance level plots. A significant difference between susceptible and resistant plots in later stages is that the recovery of many resistant barnyardgrasses in the resistant plots compensated for the death of susceptible barnyardgrasses.

The dynamics of velvetleaf herbicide stress are easier to analyze because of their individual plant growth characteristics. Velvetleaf had longer herbicide stress response times than barnyardgrass, and the susceptibility and resistance of velvetleaf were not directly related to size, as shown in Figure 6B. The mechanism of prolonged plant death generated by sink tissue toxicity in velvetleaf may be the main reason (Fuchs et al., 2002). Atrazine caused gradual inhibition of photosynthesis in velvetleaf leaves that increased over several days and was nearly complete by 5 days (Qi et al., 2018). Therefore, the difference in the spectral response of velvetleaf is smaller than that of barnyardgrass 2 days after application.

About 5 days after application, the susceptible velvetleaf began to change significantly. The NDVI images show large red leaf area reductions with relatively little activity. By contrast, the NDVI images of the resistant velvetleaf leaves changed slightly from red to yellow about 5 days after application. However, the formerly red areas began recovering about 7 days after application, sometimes even before the herbicide application, indicating that the resistant velvetleaf had resumed growth.

Both susceptible and resistant velvetleaf biotypes showed growth inhibition at the beginning of herbicide stress, but the spectral information was still better classified by inhibition differences, demonstrating the potential of spectral information for the study of resistant weeds.

### Impacts of Different Densities in Resistant Weed Classification

The distribution of barnyardgrass in a real farmland environment shows a clustered distribution (Maun and Barrett, 1986). Therefore, studies were conducted for different weed densities. The classification model was applied to different barnyardgrass densities to evaluate their reliability and adaptability. As shown in Table 5, the classification accuracy after herbicide application had maximum accuracies of 0.794 for low-density weeds, 0.736 for medium-density weeds, and 0.821 for high-density weeds. However, the velvetleaf distribution was rarely clustered, and different velvetleaf densities had almost no effect on the classification model.

**TABLE 5:** Resistant weed classification performance summary of different densities.

Densities	Metrics	2DAA	4DAA	6DAA	8DAA
Low	Accuracy	0.617	0.649	0.725	0.821
Moderate	Accuracy	0.611	0.673	0.708	0.746
High	Accuracy	0.547	0.614	0.742	0.794

**FIGURE 7:**
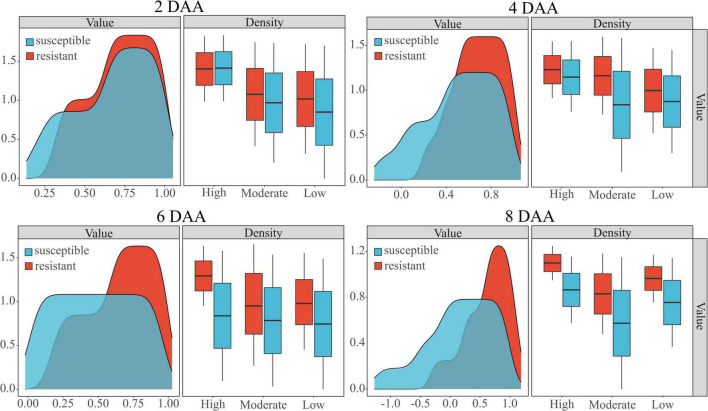
Classification of susceptible and resistant barnyardgrass biotypes at different densities (high, moderate, low) by the WSRI. DAA is days after application.

The performance of the WSRI was evaluated for each plot random samples on different densities. Figure 7 shows the WSRI density plots for susceptible and resistant barnyardgrass in the sample areas at different times after application and the box plots at different weed densities. The results show gaps between susceptible and resistant biotypes at different densities, and the gaps gradually increased over time.

In summary, atrazine spraying would encounter problems such as shading and uneven spraying under high-density barnyardgrass conditions, potentially overestimating resistance levels in susceptible areas. Moreover, symbiotic areas of resistant and susceptible barnyardgrass would affect the spectral response value, resulting in a low-classification accuracy model at early application. The low- and moderate-density areas contain few weeds. Some susceptible weeds died with increasing time after application, resulting in small fluctuations in spectral response gaps and small improvements in accuracy rates. In addition, the massive death of susceptible weeds over time in high-density areas widened the gap and improved the classification accuracy.

### Model Validation

**FIGURE 8:**
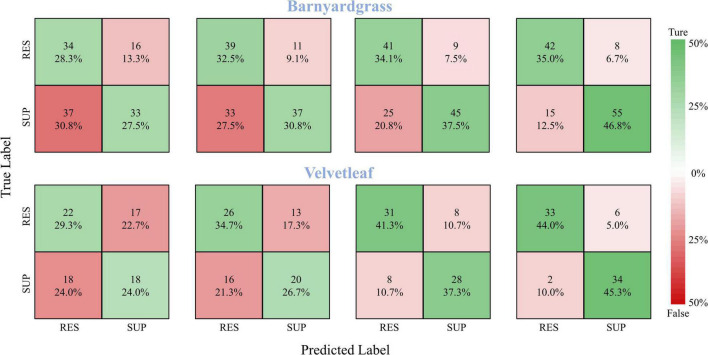
Confusion matrices for barnyardgrass and velvetleaf in the validation area. RES are resistant weeds. SUP are susceptible weeds.

A robust model should be able to generalize to new datasets and still perform well. Therefore, the model validation used the DCNN model to classify the susceptible and resistant weeds in the validation area, and the confusion matrices of the classification results are shown in Figure 8. At 8 days after application, the DCNN provided the highest classification accuracy, with 81.8% for barnyardgrass and 89.3% for velvetleaf. At 6 days after application, the DCNN provided better classification accuracy, with 71.6% for barnyardgrass and 78.6% for velvetleaf. The test results also confirmed the WSRI and DCNN model’s robustness and generality for further application.

## Discussion

### Impacts of Different Information in Resistant Weed Classification

Spectral information can reflect the physiological properties of the plant (Rajcan and Swanton, 2001), and physiological properties express differences between resistant and susceptible biotypes faster than appearance. Therefore, multispectral images can assess weed resistance faster and better than RGB images at the early stage of application. As reported in many previous works, spectral information such as VIs has become the primary remote sensing indicator for plant phenotypes because of their stable and superior performance (Ballester et al., 2017). RGB canopy structure information yielded slightly lower, but still comparable, performance than spectral information, indicating that canopy structure information is a promising alternative to commonly used VIs.

The results of this study indicate that the choice of the band is critical when establishing the vegetation index. The red and red edge bands had a significant influence on the classification of resistant weeds, and the reflectance changes of these two bands correlated with the degree of herbicide stress. The study results confirmed that the WSRI (RE-R)/(RE-B) performed well in classifying resistant weeds. The WSRI combines the effects of blue, red, and red-edge wavelengths to provide a comprehensive picture of weed dynamics after application and displayed better performance than other indices. Therefore, it provides powerful support for monitoring and investigating resistant weeds over a large canopy area using UAVs or satellites.

Time series of susceptible and resistant weed biotypes are dynamic expressions of herbicide stress. In this study, 4 days after application (4DAA) was the watershed timeframe for studying resistant weeds. The accurate timing of resistant weed investigation affects effective farmland time management. The rate of change and recovery after herbicide stress begins is key to classifying susceptible and resistant weed biotypes. Different weed species mean that differences in susceptible and resistant biotypes are expressed at different times. The classification effect of barnyardgrass was better than that of velvetleaf at the beginning of the application because of differences in their shape, physiology, and distribution characteristics. As the application time increased, the classification effect of velvetleaf became better than that for barnyardgrass.

Weed density is another factor influencing the research of resistant weeds. It is better to investigate the resistance of clustered weeds using different weed densities. The DCNN trained separately for different weed densities may increase the accuracy of susceptible and resistant barnyardgrass classifications. It is worth noting that higher densities mean the possibility of more resistant weeds, and untimely treatment multiplies the damage to the crop (Alipour et al., 2022).

### Effectiveness and Limitations of Unmanned Aerial Vehicle Traits in Resistant Weed Investigation

This study first proposed the multispectral image-derived WSRI to classify susceptible and resistant weeds in real farmland environments. For resistant weed investigation, it took at least 2 h for three raters to manually measure the distribution of resistant weeds in 40 plots. The UAV field flights took less than 15 min, which was fast enough to capture accurate data while avoiding fluctuations in environmental factors such as cloud or wind. More importantly, the high efficiency of UAV phenotyping makes dynamic monitoring with high temporal resolution possible. Therefore, UAVs have shown great potential in the emerging study of field-resistant weeds.

However, there are some limitations for the WSRI. First, changes at the early application stage may not adequately reflect the overall weed resistance because resistant weeds grow slowly during herbicide stress conditions. This is supported by the fact that differences between susceptible and resistant biotypes were not significant at the early stages, so 4 days after herbicide application is the optimal time to investigate resistant weeds. Additionally, the WSRI is an unstable measure easily affected by temperature, humidity, and light conditions in the field. Therefore, resistant weeds in the field should continue to be the subject of in-depth study and discussion.

## Conclusion

The proposed UAV–WSRI phenotypic method investigates the potential of fused multispectral and RGB image data combined with deep learning for resistant weed identification in the field. Compared with imaging chambers and expensive unmanned ground vehicle platforms, the UAV platform is more flexible and efficient to deploy for high-throughput phenotyping under field conditions. In addition, the timeliness of UAVs guarantees the reliability of phenotypic traits for resistant weed identification in the field.

The WSRI introduced in this study showed better consistency than previously published spectral VIs, with actual data for atrazine-resistant weed in maize fields. The WSRI provides better classification results than high-resolution RGB data, and the fusion of the two data types further improves the results. The robust deep learning model (DCNN) makes it possible to monitor the dynamic response to resistant weeds in the field precisely, regardless of complex environmental factors.

Our results also show that time series and weed density are closely related to resistant weed identification. The UAV–WSRI phenotypic method could be extended to evaluate the resistance response of other field weeds under herbicide stress, providing a valuable step for further field weed resistance studies.

## Data Availability Statement

The raw data supporting the conclusions of this article will be made available by the authors, without undue reservation.

## Author Contributions

FX: conceptualization, methodology, software, formal analysis, investigation, and writing—review and editing. LQ: conceptualization, writing—review and editing, supervision, and project administration. ZL and DS: visualization. HL: data curation, conceptualization, and supervision. XL: data curation. All authors have read and agreed to the published version of the manuscript.

## Conflict of Interest

The authors declare that the research was conducted in the absence of any commercial or financial relationships that could be construed as a potential conflict of interest.

## Publisher’s Note

All claims expressed in this article are solely those of the authors and do not necessarily represent those of their affiliated organizations, or those of the publisher, the editors and the reviewers. Any product that may be evaluated in this article, or claim that may be made by its manufacturer, is not guaranteed or endorsed by the publisher.

## Abbreviations

UAV: unmanned aerial vehicle
DCNN: deep convolutional neural network
VIs: vegetation index
WSRI: weed spectral resistance index
B: blue band
G: green band
R: red band
NIR: near-infrared band
RE: red edge band
RTK: real-time kinematic
GCPs: round control points
GS: Gram–Schmidt
DOM: digital orthophoto maps
BAD: before application day
AD: application day
DAA: days after application
RES: resistant weeds
SUP: susceptible weeds.
